# Combination of pan-RAF and MEK inhibitors in NRAS mutant melanoma

**DOI:** 10.1186/s12943-015-0293-5

**Published:** 2015-02-03

**Authors:** Mohammad Atefi, Bjoern Titz, Earl Avramis, Charles Ng, Deborah JL Wong, Amanda Lassen, Michael Cerniglia, Helena Escuin-Ordinas, David Foulad, Begonya Comin-Anduix, Thomas G Graeber, Antoni Ribas

**Affiliations:** Department of Medicine, Division of Hematology-Oncology, University of California Los Angeles (UCLA), Los Angeles, CA USA; Department of Molecular and Medical Pharmacology, University of California Los Angeles (UCLA), Los Angeles, CA USA; Department of Surgery, Division of Surgical-Oncology, University of California Los Angeles (UCLA), Los Angeles, CA USA; Jonsson Comprehensive Cancer Center at UCLA, Los Angeles, CA USA; New York University, New York, USA; Department of Medicine, Division of Hematology-Oncology, Jonsson Comprehensive Cancer Center, University of California Los Angeles (UCLA), 11-934 Factor Building, 10833 Le Conte Avenue, Los Angeles, CA 90095-1782 USA

**Keywords:** Melanoma, NRAS, MAPK, MEK inhibitor, pan-RAF inhibitor, Cyclin D3

## Abstract

**Background:**

Approximately 20% of melanomas contain a mutation in NRAS. However no direct inhibitor of NRAS is available. One of the main signaling pathways downstream of NRAS is the MAPK pathway. In this study we investigated the possibility of blocking oncogenic signaling of NRAS by inhibiting two signaling points in the MAPK pathway.

**Methods:**

Fourteen NRAS mutated human melanoma cell lines were treated with a pan-RAF inhibitor (PRi, Amgen Compd A), a MEK inhibitor (MEKi, trametinib) or their combination and the effects on proliferation, cell cycle progression, apoptosis, transcription profile and signaling of the cells were investigated.

**Results:**

The majority of the cell lines showed a significant growth inhibition, with high levels of synergism of the PRi and MEKi combination. Sensitive cell lines showed induction of apoptosis by the combination treatment and there was a correlation between p-MEK levels and synergistic effect of the combination treatment. Proliferation of sensitive cell lines was blocked by the inhibition of the MAPK pathway, which also blocked expression of cyclin D1. However, in resistant cell lines, proliferation was blocked by combined inhibition of the MAPK pathway and cyclin D3, which is not regulated by the MAPK pathway. Resistant cell lines also showed higher levels of p-GSK3β and less perturbation of the apoptotic profile upon the treatment in comparison with the sensitive cell lines.

**Conclusions:**

The combination of PRi + MEKi can be an effective regimen for blocking proliferation of NRAS mutant melanomas when there is higher activity of the MAPK pathway and dependence of proliferation and survival on this pathway.

**Electronic supplementary material:**

The online version of this article (doi:10.1186/s12943-015-0293-5) contains supplementary material, which is available to authorized users.

## Introduction

Over activity of the RAS/RAF/MEK/ERK mitogen-activated protein kinase (MAPK) pathway is the hallmark of the majority of melanomas, which is frequently due to mutations in *BRAF* or *NRAS* in approximately 50% and 20% of cases, respectively [1]. In melanomas with *BRAF*^*V600*^ mutation, the MAPK pathway, and therefore the growth of melanoma cells, can be efficiently blocked by BRAF inhibitors such as vemurafenib or dabrafenib [2,3]. However, no effective direct inhibitor of mutated NRAS is available.

In normal cells, RAS is the critical switch that connects the signal of activated receptor tyrosine kinases (RTKs) to the downstream signaling network particularly the MAPK pathway. In the MAPK pathway, RAF isoforms (CRAF, BRAF and ARAF) are the direct downstream proteins of RAS [4]. Upon activation, homo or heterodimers of RAF activate MEK1 and MEK2. The sole substrates of MEKs are ERK1 and ERK2, which upon activation induce activity of an array of pro-growth factors and inhibit pro-apoptotic signals [5]. In most cells, MAPK signaling is required for induction of cyclin D1 expression and therefore G1 to S phase cell cycle progression [6]. The MAPK pathway activity also induces phosphorylation of the pro-apoptotic protein BIM (BCL2L11), which targets this protein for proteasome-mediated degradation [7]. Considering the significant role of the MAPK pathway, feedback systems are in place to regulate its activity. Sprouty proteins (SPRY) negatively regulate the pathway upstream, while dual specificity phosphatases (DUSP4 and DUSP6) dephosphorylate ERK1/2 [8].

In the case of mutated RAS, the main direct effector protein is CRAF, which transfers the signal to the downstream factors in the MAPK pathway. It has been reported that CRAF also plays other roles independent of the MAPK signaling and can regulate other effectors such as MST-2 (MAP3K10) and ASK-1 (MAP3K5) [9]. There is also evidence that independent of the MAPK pathway, CRAF signaling is directly involved in regulating anti-apoptotic factors in mitochondria [10]. Despite the central role of CRAF, the signal from the mutated NRAS can be also transferred by BRAF to the downstream pathway. Studies on xenografts of a NRAS mutant human melanoma cell line indicated that shRNA knockdown of both BRAF and CRAF caused delay in the tumor formation [11]. This data indicates that perhaps a pan-RAF inhibitor (PRi) could successfully block transmission of the oncogenic signal from mutated NRAS to the downstream protein MEK.

Immediately downstream of RAFs, MEK is one of the main signaling nodes in the MAPK pathway and MEK inhibitors have shown significant growth inhibitory effects in some BRAF and NRAS mutant melanoma cells [12,13]. BRAF mutant cell lines usually show higher sensitivities, at sub-nano molar levels, to the MEK inhibitors while NRAS mutants are usually less sensitive to the inhibition of this kinase [14]. In a clinical trial with one of the MEK inhibitory drugs (MEK162) about 20% of patients with NRAS mutated melanoma showed clinical responses with a median progression free survival of 3.7 month [15]. However, the short duration of the response and progression free survival in these patients indicate that combination therapy strategies are needed to be designed for NRAS mutant melanomas. Considering the role of the MAPK signaling in induction of cyclin D1, recently a phase Ib/II clinical study with the combination of the MEK inhibitor MEK162 and a CDK4/6 inhibitor (LEE011) is being conducted (NCT01781572). Early clinical results are supportive of a potential increased antitumor effect achieved by combining a MEK inhibitor with a CDK4/6 inhibitor in patients with NRAS mutant melanoma [16].

In BRAF mutant melanomas, over-activity of alternative pathways, such as PI3K/AKT, can induce resistance to the inhibitors of the MAPK pathway [17]. Reasonably a similar mechanism of resistance may exist in some of the NRAS mutant melanomas and therefore treating them with a combination of MEKi + PI3Ki may provide beneficiary effects. Indeed, in a preclinical study, combined inhibition of MEK and PI3K/AKT pathway provided synergistic effects in NRAS mutant cells [18]. However, clinical studies are needed to determine the effectiveness of such regimens in patients.

Previous studies have shown that one of the main reasons for resistance to the targeted therapy approach is the reactivation of the main oncogenic driver pathway through the adjustment of feedback systems [19]. To prevent the reactivation of an oncogenic pathway and to achieve more effective inhibitory effects, the strategy of combination therapy with two drugs that block a pathway at two signaling points has been adopted. Along with the same line of thought, the idea of combining a BRAF inhibitor with a MEK inhibitor has been tested for the treatment of BRAF mutated melanomas. Interestingly, this regimen resulted in superior inhibitory effects and reduced toxicities [20]. However, in the case of NRAS mutant melanomas, due to the paradoxical activation of the MAPK pathway, specific BRAF inhibitors cannot be used. On the other hand, the paradoxical activation can be avoided by using a PRi that blocks activities of both BRAF and CRAF [21]. Moreover, to improve the growth inhibitory effect of the PRi in NRAS mutant melanomas and to block the feedback mechanisms that may reactivate the MAPK pathway upon the treatment with a single drug, a MEKi can be used in combination. This combination treatment may also provide the possibility of decreasing the dose of MEKi and therefore decreasing its toxicities.

In this study, we hypothesized that the oncogenic effects of mutated NRAS could be inhibited by agents that block proteins downstream of the MAPK pathway. However, to avoid the feedback or paradoxical activation of the MAPK pathway in NRAS mutated melanomas, the pathway was blocked at both RAF and MEK steps by using the combination of a PRi (Amgen Compd A) and a MEKi (trametinib). A panel of 14 human melanoma cell lines with various NRAS mutations was used for this study to investigate the growth inhibitory effect of this combination. Here, we report that combination of PRi and MEKi shows synergistic effects in the majority of NRAS mutated cell lines in the panel. Cell lines with higher activity of MAPK pathway were more sensitive to the combination therapy. The resistance to therapy and lack of synergism was the result of higher activity of pro-survival pathways and independence of cell cycle progression from the MAPK pathway.

## Results

### Synergistic effect of PRi and MEKi combination in the majority of *NRAS* mutant cell lines

**Figure 1:**
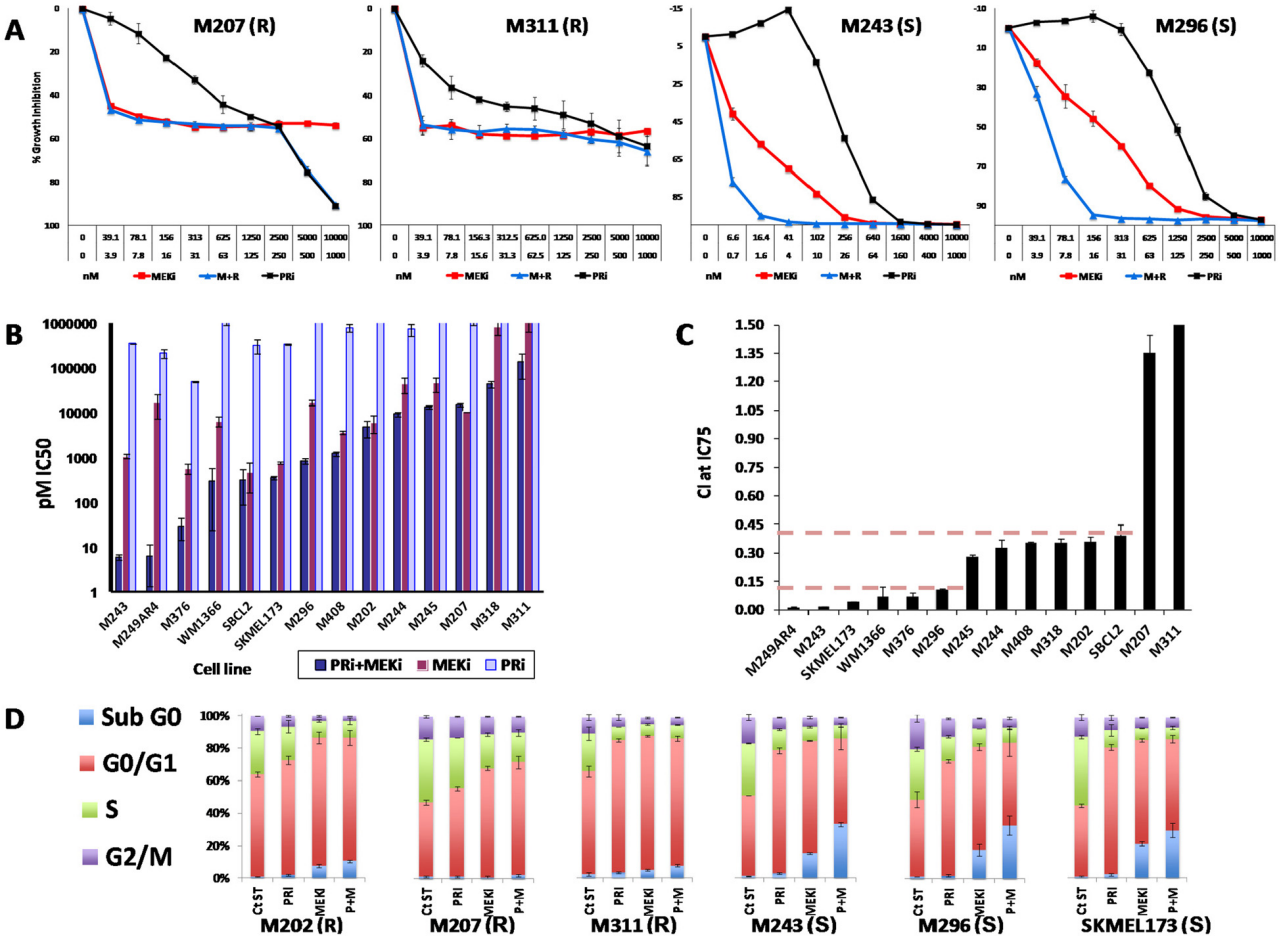
**Inhibitory effect of PRi and MEKi combination on cell growth of NRAS mutant melanoma cell lines, and effects of the combination therapy on cell cycle progression. A)** Four examples of growth inhibition assays performed on NRAS mutant melanoma cell lines. M207 and M311 are examples of cell lines resistant to both drugs and their combination. M243 and M296 cell lines are examples of cell lines with high synergistic effect of combination therapy. **B)** Bar graph of IC50s of PRi and MEKi alone or in combination. Cell lines were treated with serial dilutions of the drugs starting from the highest concentration of 10000 nM for PRi and 1000 nM for MEKi. The ratio of PRi to MEKi concentration was constant at 10:1. Cell lines are aligned in the graph according to their IC50s (in pM) of the combination treatment. **C)** Combination of PRi and MEKi shows synergistic effects in most of the tested NRAS mutant cell lines. Combination Indices (CI) at IC75 were calculated by CalcuSyn software as indicators of the synergistic effect of PRi and MEKi combination. Cell line are divided into three groups (intermittent lines), highly synergistic (CI ≤ 0.1), synergistic (0.28 < CI < 0.39) and antagonistic (CI > 1.0). **D)** Three resistant cell lines (M202, M207, M311) and three sensitive cell lines (M243, M296, SKMEL173) were treated with PRi (500 nM), MEKi (25 nM) and their combination for 48 hours. After harvest and fixation, samples were stained with DAPI for cell cycle analysis by flow cytometry. R = Resistant cell line, S = Sensitive cell line.

Growth inhibition assays were performed with single agent or a combination of PRi and MEKi on a panel of 14 *NRAS* mutant human melanoma cell lines (Table 1). Among these cell lines, M249AR4 and M376 contain both *BRAF*^*V600E*^ and *NRAS*^*Q61*^ mutations and are resistant to single agent BRAF inhibitors, and the rest have *NRAS*^*Q61*^ mutations alone (Table 1). The responses of the cell lines to single or combination treatment were variable among the cell lines (Figure 1A & B). M243 was the most sensitive cell line to the combination of PRi + MEKi treatment (IC50 = 6pM). In all the cell lines, IC50 of MEKi was lower than the IC50 of PRi. However, in some cell lines (M202, M207, M311) the MEKi growth inhibition curves showed a plateau after reaching the IC50 (Figure 1A). These cell lines also showed low responses to PRi + MEKi combination, particularly M311 which was the most resistant cell line (IC50 > 100 nM) to this treatment. In the resistant cell lines, upon reaching the plateau of growth inhibition, at some concentration points the effect of combination was slightly less than one drug alone. To identify the synergistic effect of PRi and MEKi in the panel of cell lines, Combination Indices (CI) at IC75 were calculated. Based on the CI values, cell lines were divided into three groups, six cell lines (such as M243, M296 and SKMEL173) with high synergistic (CI ≤ 0.1) effects, six cell lines with moderate synergistic effects (0.28 < CI < 0.39) and cell lines with antagonistic effects (CI > 1.0) including M207 and M311 (Figure 1C).

**Table 1:** **NRAS mutant cell lines and their characteristics**

**Cell line**	**NRAS mutation**	**Other known mutations**
**M202**	NRAS^Q61L^	EGFR amplification
		CDKN2A homozygous deletion
**M207**	NRAS^Q61L^	MITF amplification
		EGFR L747_P753 > S
		PTEN heterozygous deletion
**M243**	NRAS^Q61H^ homozygous	PTENE156G heterozygous
		CTNNB1_D32Y
**M244**	NRAS^Q61K^ heterozygous	
**M245**	NRAS^Q61K^ heterozygous	TP53R273H
**M249AR4**	NRAS^Q61K^ heterozygous	MITF amplification
	BRAF^V600E^ heterozygous	PTEN homozygous deletion
		AKT2 amplification
**M296**	NRAS^Q61R^ heterozygous	
**M311**	NRAS^Q61L^ homozygous	
**M318**	NRAS^Q61L^ heterozygous	PIK3CAC420R
**M376**	NRAS^Q61K^ heterozygous	
	BRAF^V600E^ heterozygous	
**M408**	NRAS^Q61K^ heterozygous	
**SBCL2**	NRAS^Q61K^ homozygous	
**SKMEL173**	NRAS^Q61K^	CTNNB1 D32G
		CCND1 amplification
**WM1366**	NRAS^Q61L^ heterozygous

### More pronounced effect of single agent or combination treatment on cell cycle progression of sensitive NRAS mutant cell lines

The effects of single agents or combination treatment on cell cycle progression of three resistant (M202, M207, M311) and three sensitive cell lines (M243, M296, SKMEL173) were investigated. Each cell line was treated with PRi (500 nM), MEKi (25 nM) and their combination for 48 hours. After harvest and fixation, cells were stained with DAPI for cell cycle analysis by flow cytometry (Figure 1D, Additional file 1: Figure S1). Overall, single agents or combination treatment showed more prominent effects on cell cycle progression of sensitive cell lines. Treatment with PRi induced the G0/G1 phase by 50 to 80% in the sensitive cell lines and between 16 to 32% in the resistant cell lines in comparison to their corresponding control samples. MEKi-treated sensitive cell lines showed between 16 to 21% of sub-G0 phase (an indication of apoptosis), while among the MEKi-treated resistant cell lines only one showed almost 8% of this phase. In sensitive cell lines, addition of PRi to MEKi induced the sub-G0 phase even further, showing between 42 to 118% induction in comparison with the MEKi single treatment (Figure 1D). On the other hand, in the resistant cell lines the combination treatment did not cause any further significant induction of sub-G0 phase in comparison with the MEKi single treatment. These findings are in agreement with the growth assay results indicating less drastic effects of MAPK inhibitors particularly the combination treatment on proliferation and survival of the resistant cell lines.

### Effect of single and combination treatment on signaling and feedback of the MAPK pathway in NRAS mutant cell lines

**Figure 2:**
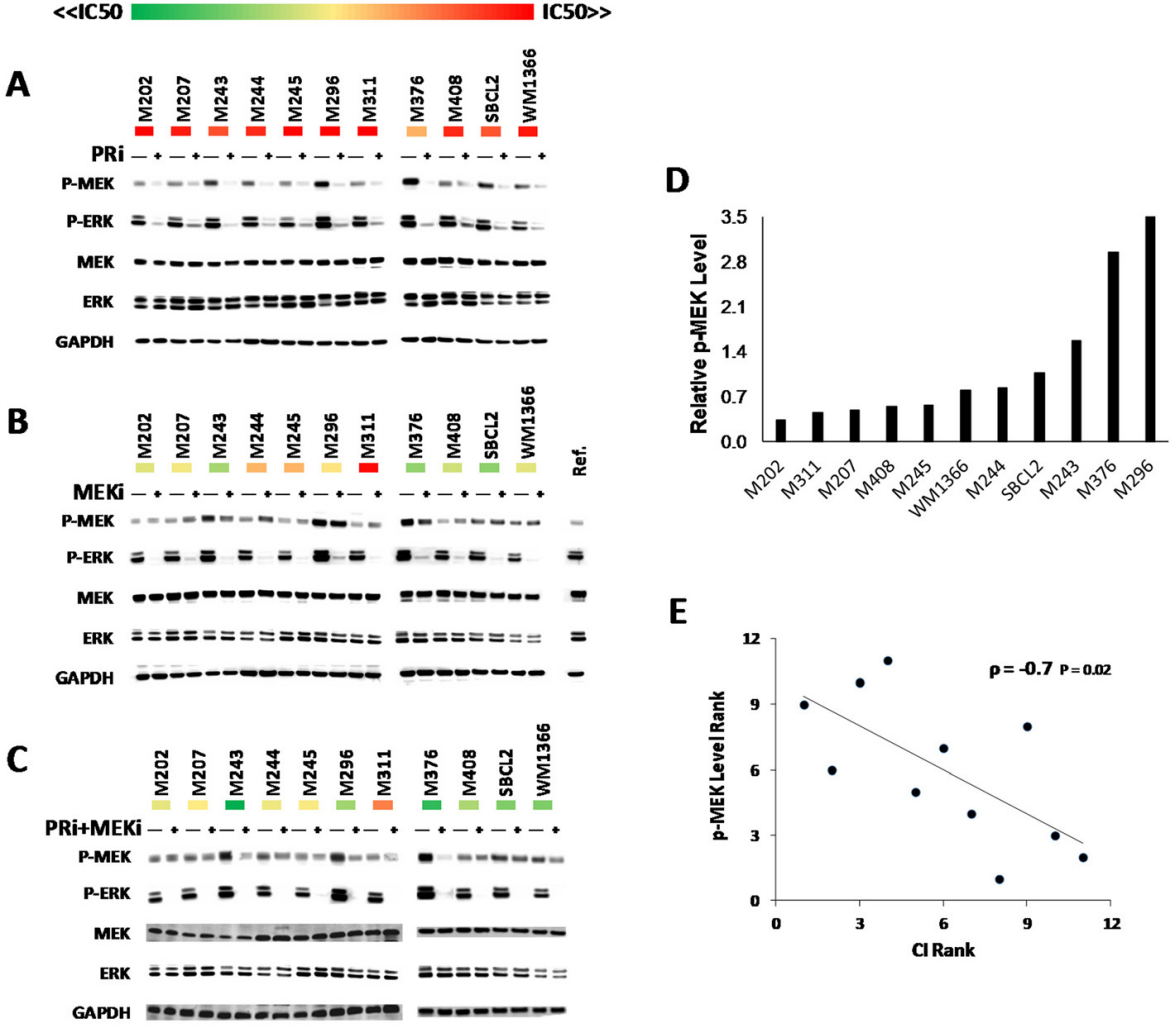
**Inhibitory effect of single agent and combination treatment on MAPK signaling and correlation of basal p-MEK level with the synergistic effect of PRi and MEKi combination. A**, **B** and **C**) Western blot analysis of NRAS mutant cell lines treated for 24 hours with DMSO, or 500 nM of PRi **(A)** or 25 nM of MEKi **(B)** or combination of 500 nM of PRi and 25 nM of MEKi **(C)**. The included heat maps were generated from log2 of IC50s (from Figure 1B) to indicate the cell lines responses across all the treatments ranging from the most sensitive (green) to the most resistant (red). **D)** Relative p-MEK levels of NRAS mutant cell lines were quantified from the intensity of untreated bands on Figure 2B. The reference band (Ref.) is the untreated M207 that was used for the normalization of values between the blots. **E)** Reverse correlation (Spearman’s Rank analysis, ρ = −0.7, p = 0.02) between the CI ranking (Figure 1C) and the ranking of p-MEK levels, indicating that the cell lines with higher levels of p-MEK exhibit higher synergistic effects with the combination treatment.

Growth inhibition assays indicated that the cell lines in the panel show variable responses to the treatments. One possible reason for this variability among the cell lines could be the result of differences in the activities of their MAPK pathway either at the baseline or after treatment with the drugs. To investigate such a possibility, NRAS mutant cell lines were treated with single agent or combination of PRi and MEKi and analyzed by Western blotting (Figure 2A, B and C). Regardless of the sensitivity or resistance of the cell lines, PRi reduced the levels of p-MEK and p-ERK. No paradoxical activation of the MAPK pathway was observed in any of the cell lines at the concentration range tested by us (Figure 2A, Additional file 2: Figure S2). As it has been described before [12], MEKi treatment caused induction of p-MEK, which was more pronounced in the resistant cell lines, and reduction in p-ERK in all the cell lines (Figure 2B). On the other hand, treatment with the combination of PRi and MEKi subsided the p-MEK inducing effect of MEKi as a single agent. Lack of p-MEK induction in the cells treated with the combination can be an indication for the interruption of the feedback or compensatory mechanisms that are induced by MEKi single agent treatment. In comparison with the single agent treatment, combination of PRi + MEKi caused even further decreases in p-ERK levels of all the cell lines to below the detectable level (Figure 2C). It seems that this combination is more effective for the complete block of the MAPK pathway.

### Correlation of p-MEK level with synergistic effect of PRi + MEKi

The baseline activity of MAPK pathway was variable among the tested cell lines. Some of the more sensitive cell lines to the combination therapy showed higher basal levels of p-MEK and p-ERK than the more resistant cell lines. To investigate the association of baseline MAPK pathway signaling with the sensitivity to the treatment, the baseline levels of p-MEK for each cell lines was quantified by densitometry of the Western blots, as it has been described in the methods and materials section. After normalization and ranking of the p-MEK levels (Figure 2D), by performing Spearman’s Rank analysis, we found a significant inverse correlation (ρ = −0.7, p = 0.02) between the ranking of p-MEK levels and ranking of Combination Indices at IC75s of combination treatment (Figure 2E, Additional file 3: Figure S3, Additional file 4: Figure S4). This correlation indicates that the cell lines with higher levels of p-MEK, or higher activity of MAPK pathway, exhibit higher synergistic effects with the combination treatment and vice versa. These findings suggest that perhaps the higher level of MAPK activity is an indication for the higher dependency on this pathway and therefore higher sensitivity to the complete blockade of this pathway.

### Expression profiling of resistant and sensitive cell lines indicating similar MAPK, but distinct cell-cycle and apoptosis response to the treatments

**Figure 3:**
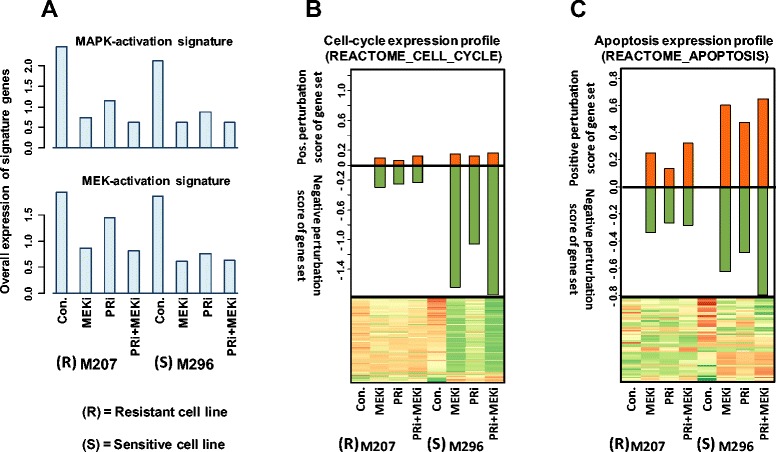
**Expression profiles of M207 and M296 indicating a similar MAPK, but distinct cell-cycle and apoptosis response to the treatment. A)** Analysis of MAPK signaling by gene expression profiles of resistant cell line M207 and the sensitive cell line M296. **B)** The effect of treatments on cell cycle expression profile of M207 and M296 are depicted by the heat map and the bar graph. **C)** Higher perturbation of apoptotic profile by the treatments in the sensitive cell line M296 in comparison with M207. For these experiments cell lines were treated with DMSO, 25 nM of MEKi, 500 nM of PRi or their combination for 24 hours before extraction of RNA for the microarray analysis.

In order to better understand the mechanism of sensitivity or resistance, we performed transcription microarray analysis on M207 and M296 cell lines, which are representative of the cell lines with no synergistic or highly synergistic responses to combination treatment, respectively. As we investigated the effect of the treatments on the published signatures of MAPK-activation and MEK-activation [8,22], we observed a similar pattern of down regulation of these signatures in both cell lines (Figure 3A). These results are in agreement with Western blot data (Figure 2). Since in both cell lines the MAPK signature was down regulated by the treatments, perhaps the resistance of M207 is due to the lower dependency of this cell line on MAPK pathway or the result of activities of pathways other than the MAPK.

One of the main differences between M207 and M296 was in the effect of treatments on the cell cycle expression profiles, which is in agreement with the phenotypes observed in cell cycle analysis experiments (Figure 1D). As it is indicated by the heat map and the bar graph in figure 3B, blocking of MAPK pathway caused the overall down regulation of cell cycle profile more prominently in M296 than in M207. The down regulation of the cell cycle profile was not confined to a particular phase of cell cycle. In the sensitive cell line M296, in addition to cyclin D1, that is involved in G1 to S phase progression, other factors involved in the subsequent phases such as CDK1, CDK2, cyclin A2 and cyclin B1 were also down regulated by the treatments.

Moreover, in comparison with the resistant cell line M207, the apoptosis response profile of M296 showed higher perturbations with the treatment (Figure 3C). In M296, higher perturbation of apoptotic profile by the treatments, particularly the combination treatment, may reflect an overall shift of balance from survival to apoptosis in this sensitive cell line. This is particularly more evident through the lower expression of some anti-apoptotic factors such as BCL2L1 and BCL2L12 and the increase in expression of pro-apoptotic factors such as BCL2L11 (BIM) in treated M296 cells. In this cell line, this perturbation pattern is perhaps due to the direct or indirect dependence of these factors expression on the MAPK pathway. Considering the microarray results regarding the cell cycle progression and apoptosis, we sought to perform further experiments at protein and cellular levels to investigate the role of important cell cycle progression factors in sensitivity or resistance. Moreover, since apoptosis or survival is concerted through the intricate balance of pro and anti-apoptotic factors at protein level, in the following experiments, we investigated the presence of pro-survival signaling, effect of treatment on BCL2L11 protein level and induction of apoptosis at cellular level.

### Lack of dependency of resistant cell lines on cyclin D1 and their dependency on cyclin D3 for their proliferation

**Figure 4:**
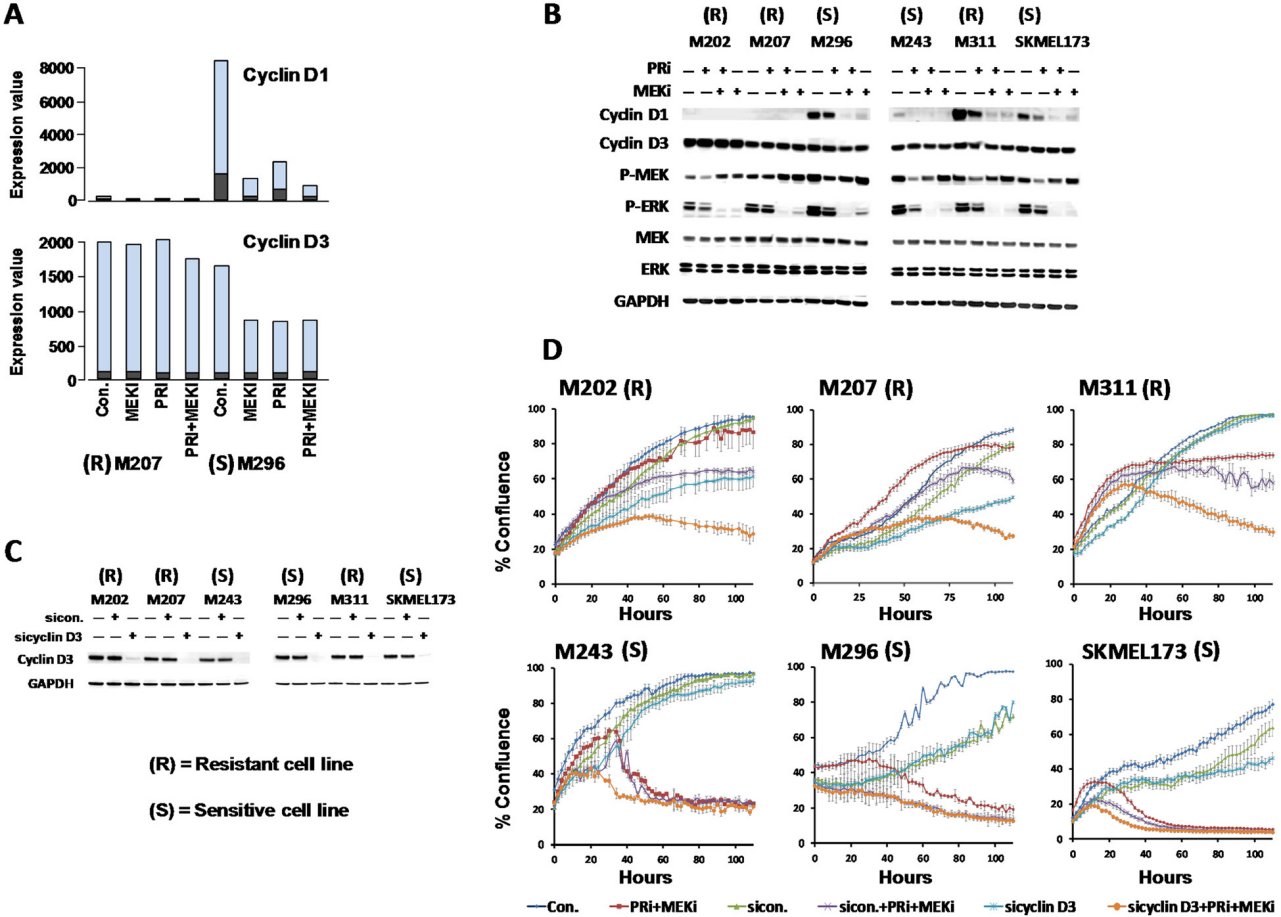
**Negative growth in resistant cell lines upon knockdown of cyclin D3 and blockade of the MAPK pathway. A)** Bar graphs of cyclin D1 and cyclin D3 RNA expression in M207 and M296 cell lines determined by transcription microarray analysis as it was described before. Different colors on the bars are the representation of signals from different probes for the same gene. **B)** Western blot analysis of cyclin D1 and D3 expressions in three resistant (M202, M207, M311) and three sensitive (M243, M296, MSKMEL173) cell lines treated for 24 hours with 500 nM of PRi, 25 nM of MEKi or their combination for 24 hours. **C)** Western blot analysis indicating successful blockade of cyclin D3 expression 72 hours after the transfection of the cells with a cyclin D3 specific siRNA pool. **D)** Effect of cyclin D3 knockdown on growth of resistant and sensitive cell line. In this experiment, kinetics of growth was monitored and recorded by an InCucyteZOOM device during a period of 110 hours. In the resistant cell lines (upper panel), negative growth was achieved by the combination of cyclin D3 knockdown + PRi + MEKi. All the conditions were in triplicates and the assay was repeated twice.

The microarray analysis indicated an overall down regulation of cell cycle profile upon treatment of the sensitive cell line M296. One of the important factors in this profile is cyclin D1, which interacts with CDK4/CDK6 and regulates transition of cell cycle from G1 to S phase. It is known that expression of cyclin D1 is regulated by activity of the MAPK pathway [6]. Expectedly, expression of cyclin D1 RNA showed drastic decreases in M296 treated with the inhibitors of MAPK pathway (Figure 4A). However, regardless of the presence or absence of the drugs, RNA of cyclin D1 was not detected in the resistant cell line M207 (Figure 4A). On the other hand, RNA expression of another member of cyclin D family, cyclin D3, was detected in both cell lines. It is been suggested that the expression of cyclin D3 is not directly regulated by the activity of MAPK pathway [23]. Accordingly, expression of cyclin D3 was not affected by the MAPK inhibitors in M207, however treated M296 samples showed some reduction. No RNA expression of cyclin D2 was detected in either cell lines.

These findings were further investigated at the protein level by Western blot analysis in three resistant (M202, M207, M311) and three sensitive (M243, M296, MSKMEL173) cell lines. No band for cyclin D1 was detected in M202 and 207 confirming the RNA expression data (Figure 4A, B and not shown RNA sequencing data). In other cell lines, including the resistant cell line M311, expression of cyclin D1 was down regulated by the inhibitors of the MAPK pathway, particularly by the combination of the drugs (Figure 4B). Cyclin D3 protein was detected in all the cell lines and the resistant cell line M202 showed the highest expression of this protein. Levels of this protein were not affected by the MAPK inhibitors except a very slight decrease in M296 cell line.

Considering these findings, we hypothesized that the proliferation of resistant cell lines is perhaps dependent on cyclin D3 and therefore to some extend should be independent of MAPK pathway. To test this hypothesis, expression of cyclin D3 was successfully blocked by a cyclin D3-specific siRNA pool (sicyclin D3) in all the six tested cell lines (Figure 4C). The proliferation kinetics upon the knockdown of cyclin D3 in the presence and absence of complete blockade of the MAPK pathway by PRi + MEKi treatment was monitored and recorded by automated imaging analysis (Figure 4D). In the resistant cell lines M202, M207 and M311 (Figure 4D upper panel), negative growth rates were achieved only by the treatment with the combination of sicyclin D3 + PRi + MEKi, which reflects their proliferation dependency on cyclin D3. This includes M311 cell line that despite its high baseline expression of cyclin D1, and unlike the sensitive cell lines, requires the combination of cyclin D3 knockdown and blocking of the MAPK pathway (which also blocks cyclin D1 expression) to show negative growth rate. On the other hand, the sensitive cell lines (lower panel) showed negative growth by the combination of PRi + MEKi regardless of the cyclin D3 knockdown. Overall, these data indicate that one of the mechanisms of resistance is the dependency of the resistant cell lines on cyclin D3, which is not mainly regulated by MAPK pathway and cannot be down regulated by the inhibitors of this pathway.

### High endogenous activity of pro-survival signaling and lower apoptotic response of resistant cell lines to the inhibition of MAPK pathway

**Figure 5:**
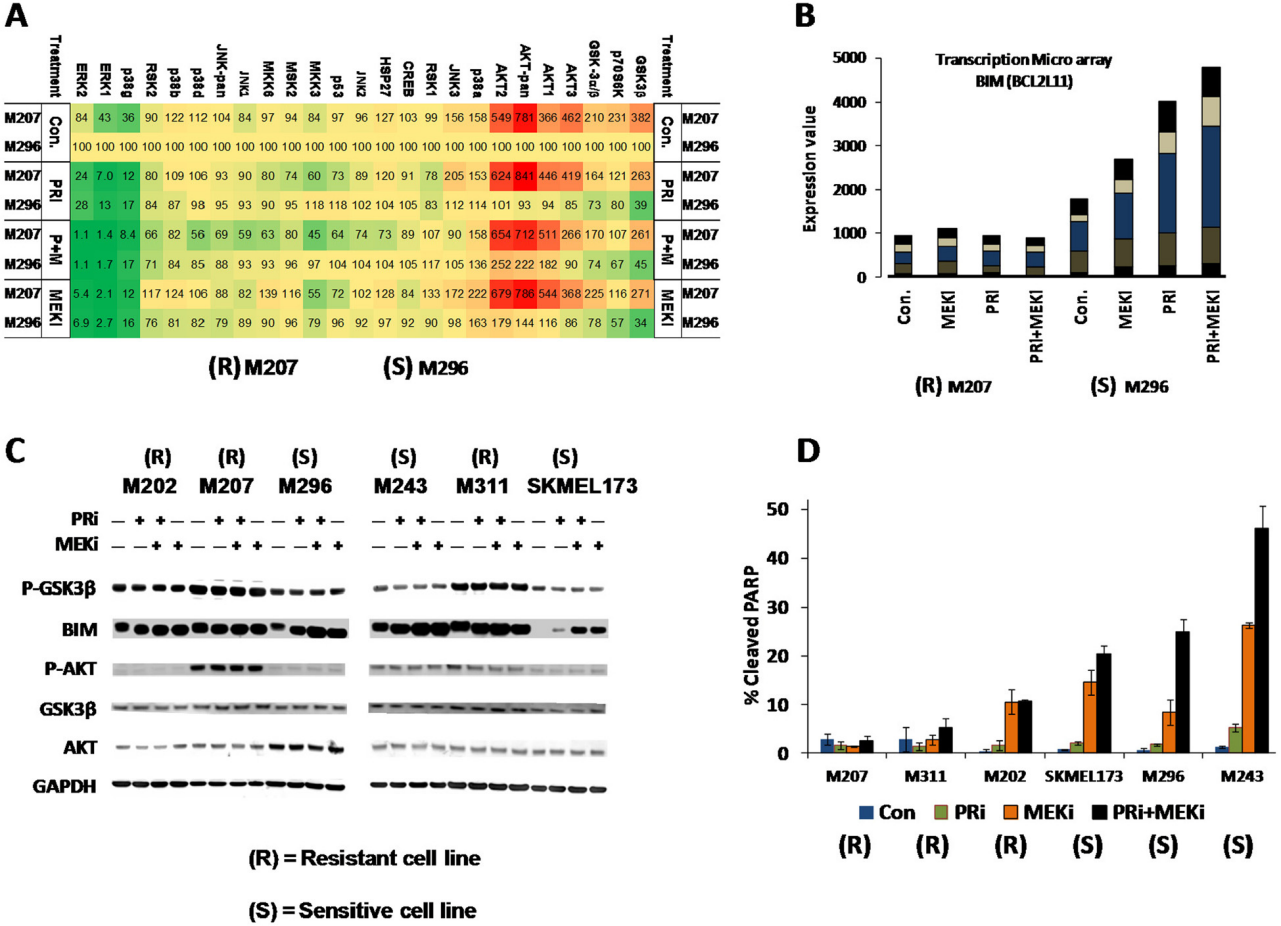
**High endogenous activity of pro-survival signaling and low apoptotic response of resistant cell lines to the blockade of MAPK pathway. A)** Higher levels of p-GSK3β and p-AKT in the resistant cell line M207, determined by antibody array analysis of 24 phosphoproteins. M207 and the sensitive cell line M296 were treated with the solvent, PRi, MEKi or their combination for 24 hours. The signal from solvent treated M296 was used as the reference (indicated as the value of 100) for all other samples. **B)** Induction of BIM RNA expression by the MAPK inhibitors, in the M296 but not in M207 determined by transcription microarray analysis. Different colors on the bars are the representation of signals from different probes for the same gene. **C)** Western blot analysis of three resistant cell lines (M202, M207, M311) and three sensitive cell lines (M243, M296, SKMEL173) indicating higher levels of p-GSK3B in the resistant cell lines and higher fold induction of BIM in sensitive cell line upon the treatments for 24 hours. **D)** Higher apoptotic response of the sensitive cell lines to the combination treatment determined by cleaved PARP flow cytometry assay. Cells were treated with PRi (500 nM), MEKi (25 nM) or the combination for 48 hours before the harvest, fixation and antibody staining.

In order to identify the signaling mechanism of resistance to the combination treatment, we performed antibody array analysis in the resistant cell line M207 and the sensitive cell line M296 (Figure 5A, Additional file 5: Figure S5). In agreement with the previous Western blotting assays, the antibody array also showed that at the baseline level, ERK proteins were less phosphorylated in M207 than M296.

Based on the antibody array results, the most prominent differences between these two cell lines, particularly after the treatment with PRi + MEKi, were the higher levels of pro-growth and pro-survival factors, such as p-GSK3β and p-AKT in the M207 cell line (Figure 5A). Antibody array also indicated a consistent and further decrease in p-GSK3β upon treatment of the sensitive cell line M296. Contrary to this finding in the M207 cell line, as it was shown before by the transcription microarray, the treatments caused higher perturbation of the pro-apoptotic profile in M296 (Figure 2C). Of note, RNA expression of the pro-apoptotic gene *BCL2L11* (BIM) was induced by the MAPK inhibitors, particularly the combination treatment, in M296 but not in M207 cell line (Figure 5B).

To confirm these findings, we investigated levels of p-GSK3β, BIM and p-AKT in representative resistant and sensitive cell lines (Figure 5C). Interestingly, we identified two patterns that distinguished the resistant cell lines (M202, M207, M311) from the sensitive ones (M243, M296, SKMEL173). First, p-GSK3β levels were higher in the resistant cell lines in comparison to the sensitive lines. Second, although the baseline protein levels of BIM were lower in the sensitive cell lines, blocking of MAPK pathway caused significant induction of this protein in comparison with the basal BIM levels. In the resistant cell lines, relative to the BIM basal levels, expression of this protein was either not induced by the treatment or the relative induction was not to the same magnitude observed in the sensitive cell lines. Although p-AKT was higher in the resistant cell line M207, we did not find a distinguish pattern of AKT phosphorylation among the rest of cell lines.

All these results referred to the higher levels of pro-survival factors and less perturbations of pro-apoptotic factors in the resistant cell lines. Indeed, as we investigated the effects of PRi and MEKi on induction of apoptosis in the sensitive cell lines versus the resistant ones, the results indicated that the combination treatment, for 48 hours, caused higher levels of cleaved PARP (20% to 46%) in the sensitive cell line (Figure 5D). Moreover, in the sensitive cell lines addition of PRi to MEKi enhanced the apoptotic effect of MEKi alone by 1.4 to 2.9 fold. On the contrary, in the resistant cell lines treatments with the same drugs induced less than 11% apoptosis. Overall, these results provide strong evidence for the presence of pro-survival and anti-apoptotic signaling in the resistant cell lines which play important roles in diminishing the effect of complete blockade of the MAPK pathway by the combination treatment.

## Discussion

NRAS mutation is detected in around 20% of melanomas; however no effective direct inhibitor of mutated NRAS is clinically available [1]. The MAPK pathway is one of the most important pathways downstream of mutated RAS [4]. Therefore, we investigated the effect of inhibiting the MAPK pathway as an alternative way of blocking oncogenic signaling of mutated NRAS. It is necessary to consider that: i) RAS mutants are less sensitive to the MEK inhibitors in comparison to the BRAF mutant melanomas [14]; ii) NRAS mutants exhibit paradoxical activation of the MAPK pathway upon the treatment with the BRAF inhibitors such as vemurafenib [21]; and iii) there is possibility of reactivation of the pathway trough the adjustment of feedback systems. Our strategy for avoiding or minimizing these issues was to block the MAPK pathway at two signaling points by using the combination of a RAF inhibitor, which blocks CRAF and BRAF simultaneously and prevents paradoxical activation, with a potent MEKi. Indeed, in this study the use of the PRi in combination with the MEKi provided a complete shutdown of the MAPK pathway in all the tested cell lines. The majority of the cell lines showed a significant growth inhibition and high levels of synergism of these two drugs. However, despite the complete blockade of MAPK pathway signaling, some cell lines showed resistance to single agent and the combination of the drugs, and exhibited low or lack of synergism.

Interestingly, we found a variable baseline activity of MAPK pathway among the cell lines in the NRAS mutant panel. Sensitive cell lines showed higher level of p-MEK and p-ERK as opposed to the more resistant cell lines that had lower baseline levels of these two phosphoproteins. Indeed, we found a significant correlation between the ranking of p-MEK levels and the synergistic effect of complete blocking of the MAPK pathway by the combination treatment. These findings suggest that perhaps higher activity of the MAPK pathway is an indication for higher dependency of the cells on this pathway and therefore higher sensitivity of these cell lines to the blockade of the MAPK pathway.

In addition, upon performing cell cycle analysis and apoptosis assays we observed a distinguished pattern of response to the drugs in sensitive cell lines versus the response of resistant cell lines. In most of the cells, progression from G0/G1 to S phase is regulated by the interaction of one of the cyclin D isoforms with CDK4/CDK6. Indeed the effect of the MAPK pathway on induction of cell cycle progression is mainly through the induction of cyclin D1 expression [6]. Interestingly in two of the resistant cell lines we did not detect expression of cyclin D1 and the only expressed isoform was cyclin D3. Expression of cyclin D3 is believed to be independent of the MAPK pathway activity [23]. Considering these results, we tested the hypothesis that the cell cycle progression of resistant cell lines is dependent on cyclin D3 and therefore is independent of MAPK pathway signaling. Indeed, by a combination of siRNA to cyclin D3 + PRi + MEKi, we achieved a negative growth in the three tested resistant cell lines while in the sensitive cell lines combination of PRi + MEKi was sufficient to induce negative growth rate. Therefore, we concluded that one of the main mechanisms of resistance to this combination is the dependency of cell cycle progression on cyclin D3, which is not regulated by the MAPK and hence cannot be blocked by the inhibitors of this pathway.

Both cyclin D1 and D3 can bind to CDK4/6 to induce cell cycle progression from G1 to S phase. To block the function of these cyclin-CDK complexes, inhibitors of CDK4/6 have been developed. While in theory these inhibitory compounds should be effective in cancer cells that are dependent on either one of the cyclin Ds (cyclin D1, D2 or D3), it is not clear if in practice they show such a general effect. For instance, in an *in vitro* study on breast cancer cell lines, the sensitivity to the CDK4/6 inhibitor palbociclib (PD 0332991) was mainly limited to the luminal cell lines with a microarray signature of high cyclin D1, high RB and low CDKN2A; meanwhile cyclin D3 was not part of the sensitivity signature [24]. In the case of NRAS mutant melanomas, a recent phase Ib/II clinical study with the combination of the MEK inhibitor MEK162 and a CDK4/6 inhibitor (LEE011) is being conducted (NCT01781572). Early clinical results are supportive of a potential increased antitumor effect achieved by combining a MEK inhibitor with a CDK4/6 inhibitor in patients with NRAS mutant melanoma [16].

In addition to the cell cycle progression, our results also indicated that resistant cell lines are better equipped to support their survival independent of the MAPK pathway. The role of GSK3β in regulation of different cell functions including metabolism, cell cycle progression and cell survival has been very well studied. Phosphorylation of GSK3β inhibits its activity and promotes cell proliferation and survival [25]. Interestingly, in our study one of the main differences between the resistant and sensitive cell lines was the higher levels of p-GSK3β in the resistant cell lines. Moreover, inhibition of the MAPK showed no significant effect on the level of this phosphoprotein in the Western blot analysis of the resistant cell lines.

Usually, p-GSK3β is considered to be downstream the PI3K/AKT pathway. In this study, although higher levels of p-GSK3β were detected in all three resistant NRAS mutant cell lines, high levels of p-AKT was not detected in two of them (Figure 5C). In these PRi + MEKi resistant cell lines, presence of such pro-survival factor independent of AKT, and the cyclin D3 dependent proliferation, may argue for a the possibility of a concomitant resistance to the inhibitors of PI3K/AKT pathway as well. Although in some studies on NRAS mutant cells, synergistic effect of combining MEKi and inhibitors of PI3K/AKT have been observed [18], further pre-clinical and clinical studies are required to determine the effectiveness of such combinations in the resistant cases.

One of the pro-survival effects of ERK is exerted through the phosphorylation of the pro-apoptotic protein BIM and therefore targeting this protein for degradation. Interestingly, in comparison with the basal BIM levels, blockade of MAPK pathway caused relatively higher fold inductions of BIM protein in the sensitive cell lines. This is an indication for the higher perturbation of pro-apoptotic factors and the shift of balance toward apoptosis upon the blockade of the MAPK pathway in the sensitive cell lines. Indeed, in transcriptome studies, we observed the high perturbation in the pro- apoptotic profile of treated sensitive cell line, M296. As it was expected, by performing cleaved PARP apoptosis assays, we observed higher rates of apoptosis in PRi + MEKi treated sensitive cell lines as compare with the resistant ones.

As it has been shown in BRAF mutant melanomas, resistance to blocking of the MAPK pathway can be the result of reactivation of the same pathway or activation of alternative pathways. Reasonably the same pattern of endogenous or developing resistance can exist in NRAS mutant melanomas. In this study, we observed the synergistic effect of PRi + MEKi combination in majority of NRAS mutant cell lines particularly in those with higher activity of the MAPK, but not in cell lines with MAPK independent pro-survival and cell cycle progression factors. In the future preclinical and clinical studies, it would be interesting to find out whether this combination shows better outcomes and delays the onset of resistance in comparison to single agent MEKi.

## Conclusion

Considering the findings of this study, our general conclusion is that level of basal p-MEK in each cell line reflects the activity and importance of the MAPK pathway in the cells. Perhaps, cell lines with higher levels of p-MEK are also more dependent on this pathway and more sensitive to the blockade of this pathway and its consequences such as down regulation of pro-survival and cell cycle progression factors such as cyclin D1. On the other hand, cell lines with lower activities of the MAPK pathway, that contain higher activities of other pro-survival pathways such as high p-GSK3β and dependency on cyclin D3 for cell cycle progression are resistant to the blockade of the MAPK pathway by the combination treatment.

## Materials and methods

### Reagents and cell lines

Trametinib was purchased from Selleck Chemicals (Houston, TX) and the pan-RAF inhibitor (Amgen Compd A - hereafter PRi) [26] was obtained from Amgen (Thousand Oaks, CA) under a materials transfer agreement (MTA). Human melanoma cell lines (M series) were established from patient’s biopsies under UCLA IRB approval # 11–003254. WM1366, SKMEL173 and SBCL2 cell lines were obtained from the American Type Culture Collection (ATCC, Rockville, MD). Cells were maintained and tested for mycoplasma as described before [17]. Presence of mutations in the genes of interest was checked by OncoMap 3 or Iontrone, and was confirmed by PCR and Sanger sequencing.

### Cell proliferation and viability assays

Melanoma cell lines were treated with serial dilutions of trametinib, PRi, their combinations or DMSO for 72 hours or 5 days depending on the growth rate of the cell lines. The assays were performed in duplicates for each concentration and repeated at least twice. Cell viability was detected by a bioluminescence assay (Promega, Madison, WI). The IC50s were calculated from the growth inhibition assays. To determine synergistic effect of the drugs, Combination Index (CI) was calculated by CalcuSyn software (version 2.0 Biosoft, Cambridge, UK).

### siRNA transfection and recording of growth kinetics

Cell lines were transfected with the cyclin D3 specific or no target siRNAs (Dharmacon, Lafayette, CO), as it has been described before [17] and were cultured in 96 well plates. After 24 hours the cells were treated with DMSO or PRi (500 nM) + trametinib (25 nM) combination. The growth rate in each well was continuously monitored and recorded by IncuCyteZoom instrument (Essen BioScience, Ann Arbor, MI) in a period of 110 hours. Each assay was performed in triplicate and repeated at least twice.

### Western blotting and quantification of signal intensities

Western blotting was performed as previously described [17]. Primary antibodies included p-AKT Ser473, AKT, p-ERK Thr204/205, ERK, p-MEK Ser217/221, MEK, cyclin D1, cyclin D3, p-GSK3β, GSK3β, BIM and GAPDH (all from Cell Signaling Technology, Danvers, MA). The immunoreactivity was revealed by use of an ECL2 kit (Pierce Rockford, IL) and scanning of the blots by the Typhoon scanner (Amersham Biosciences Co, Piscataway, NJ). The intensity of the p-MEK bands were determined by the ImageQuant software and were normalized between the two blots by considering the reference sample (one of the samples of the first blot duplicated on the 2nd blot). The relative level of p-MEK for each sample was determined by the ratio of each sample over the average intensity of all the samples.

### Cell cycle analysis and apoptosis

Cell lines were treated with DMSO or 500 nM PRi, 25 nM of MEKi or their combinations for 48 hours. After the harvest, cell cycle analysis was performed as it was described before [12]. For analysis of apoptosis, cells were treated, harvested, fixed and prepared similar to the cell cycle analysis and then stained for detection of cleaved poly [ADP-ribose] polymerase (PARP) by anti–PARP-Alexafluor700 (clone F21-852; BD Biosciences). All flow cytometry experiments were performed on LSRII (BD Biosciences) flow cytometry machine [27].

### Transcription microarray analysis

M207 and M296 cells were treated for 24 hours with 500 nM PRi, 25 nM of MEKi, or their combination. After harvest total RNA was isolated by RNeasy kit (Qiagen, Valencia, CA). Further processing, hybridization (Affymetrix Human U133plus2.0 Array), and slide scanning were performed by the Clinical Microarray Core at UCLA. The data was processed with R/Bioconductor [28] and samples were RMA normalized. To calculate the MAPK-activation and MEK-activation signature responses the respective gene sets were obtained from Pratilas *et al.* [8] and Dry *et al.* [22]. The expression data was log-transformed, centered, and scaled. The gene expression of the signature genes was summarized as their median expression value. The cell-cycle and apoptosis gene sets were obtained from the Reactome database (“REACTOME_CELL_CYCLE”, “REACTOME_APOPTOSIS”). The expression data for the subsets was filtered for a two-fold up- or down-regulation in either cell line (combination treatment vs. control). For the heat map visualization the data was log-transformed and scaled. The overall effects of the treatments for cell-cycle and apoptosis gene sets were determined using log-transformed data, and the difference of expression between the treated and untreated samples was calculated for each cell line. Multiple probes for each gene were collapsed to their average. The positive (or negative) perturbation score (bar graph) was calculated as the sum of the positive (or negative) relative expression values divided by the total number of genes in the expression gene set.

### Human Phospho-MAPK array analysis

Detection of a panel of phosphoproteins in M207 and M296 was carried out by treating them with DMSO or PRi (500 nM), MEKi (25 nM) or combination of the drugs for 24 hours and using Human Phospho-Kinase Array Kit (R&D Systems, Inc., Minneapolis, MN). Harvest of cells, lysis and all other steps were performed according to the instruction of the manufacturer. To detect the signal levels, arrays were exposed to ECL2 reagent (Pierce Rockford, IL), scanned and signals were quantified as it was described in the Western blotting in above. The signals were normalized between the arrays by considering the reference spots. The relative signal level for each phosphoprotein was determined by calculating the ratio of each signal over the signal of M296 control which was assigned the value of 100.

## Additional files

Additional file 1: Figure S1. Flow cytometry histograms of cell cycle analysis of 3 resistant and 3 sensitive cell lines treated for 48 hours with PRi, MEKi or their combination indicating a significant induction of sub-G0 phase in the sensitive cell lines upon the treatment with combination of PRi + MEKi. Additional file 2: Figure S2. Inhibitory effect of different concentrations of PRi, single dose MEKi and PRi + MEKi on activity of the MAPK pathway signaling. Cells were treated for 24 hours with the mentioned conditions. Additional file 3: Figure S3. CI75 and p-MEK level correlation plot in NRAS mutated cell lines (Spearman’s rank correlation analysis and Pearson’s product moment correlation). Ranking of CI75 of PRi + MEKi shows an inverse correlation with the ranking of p-MEK levels. Additional file 4: Figure S4. IC50 and p-MEK level correlation plots in NRAS mutated cell lines (Spearman’s rank correlation analysis). Ranking of IC50s of PRi + MEKi shows an inverse correlation with the ranking of p-MEK levels. **A)** Correlation plot of ranking of PRi IC50s and ranking of p-MEK levels. **B)** Correlation plot of ranking of MEKi IC50s and ranking of p-MEK levels. **C)** Correlation plot of ranking of PRi + MEKi IC50s and ranking of p-MEK levels. Additional file 5: Figure S5. Human Phospho-MAPK Array for detection of 24 phosphoproteins performed on melanoma cell lines. **A)** Differences in signaling of the resistant and sensitive cell lines M207 and M296 were investigated after treatment of these cell lines with the solvent, PRi (500 nM), MEKi (25 nM) and their combination for 24 hours. For this purpose the antibody array from R & D Systems was used which detects 24 phosphoproteins in duplicates. Each spot represent one phosphoprotein. The signals from the Reference spots were used for the normalization among the arrays. **B)** Map of the phosphoprotein spots on the array adopted from R & D systems website.
