# Supplementary figures and images for: Combination of pan-RAF and MEK inhibitors in NRAS mutant melanoma
Source: Mol Cancer. 2015 Feb 3;14(1):27. doi: 10.1186/s12943-015-0293-5 (PMC4320814; doi:10.1186/s12943-015-0293-5)

## Slide 1
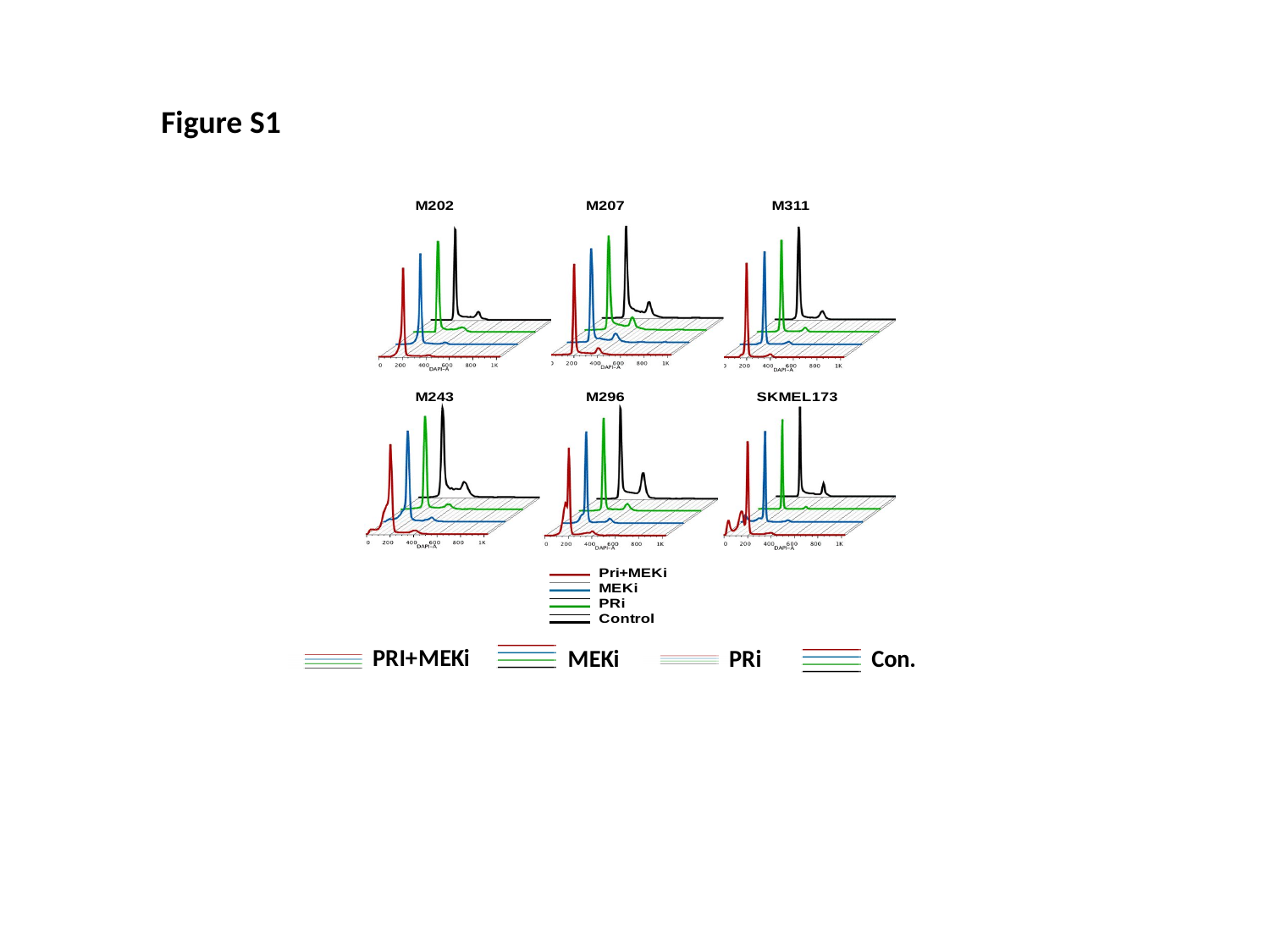

Figure S1

Supplement: Additional file 1: Figure S1. — Flow cytometry histograms of cell cycle analysis of 3 resistant and 3 sensitive cell lines treated for 48 hours with PRi, MEKi or their combination indicating a significant induction of sub-G0 phase in the sensitive cell lines upon the treatment with combination of PRi + MEKi. [file 12943_2015_293_MOESM1_ESM.ppt]

## Slide 1
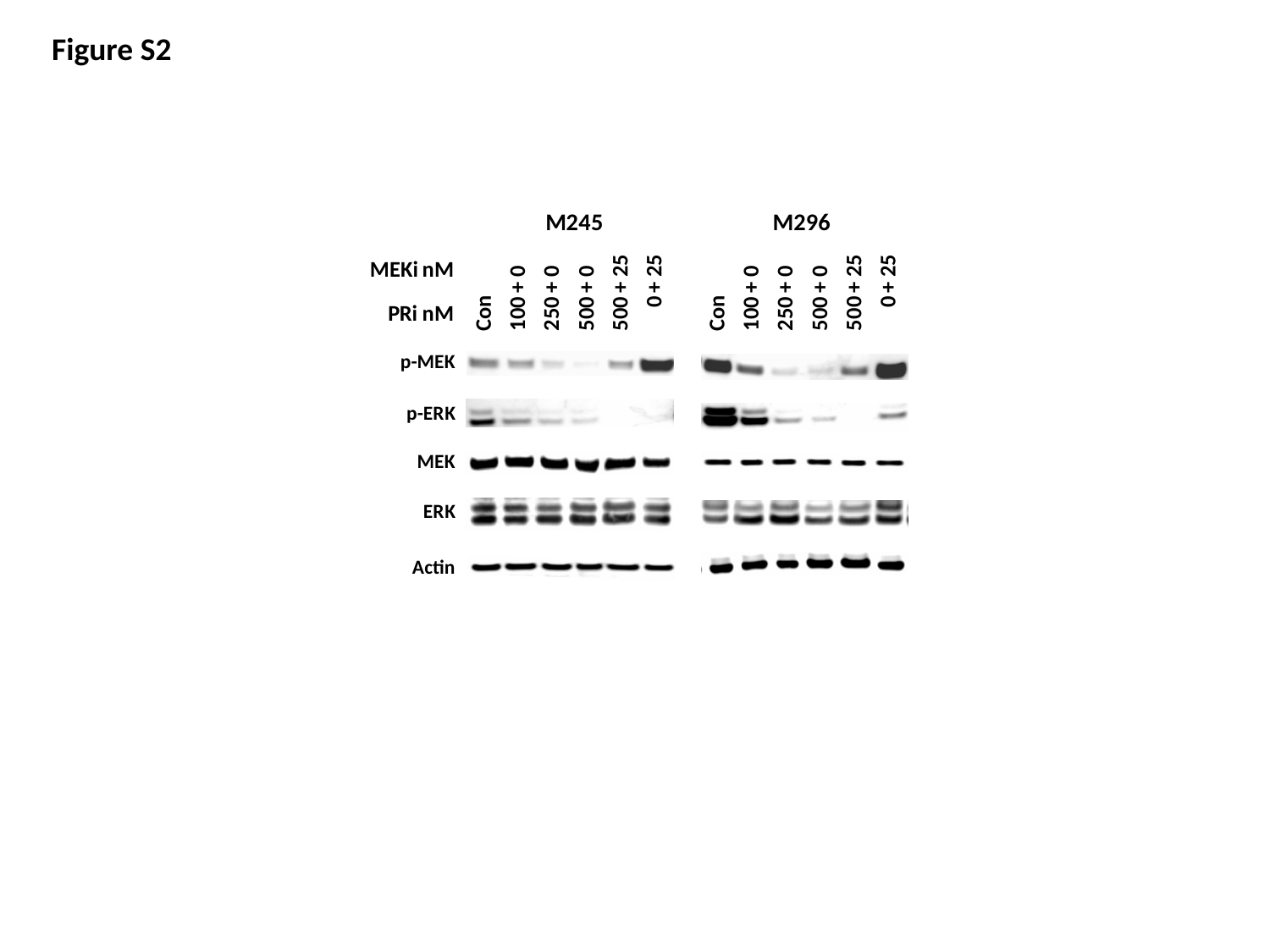

Figure S2

Supplement: Additional file 2: Figure S2. — Inhibitory effect of different concentrations of PRi, single dose MEKi and PRi + MEKi on activity of the MAPK pathway signaling. Cells were treated for 24 hours with the mentioned conditions. [file 12943_2015_293_MOESM2_ESM.ppt]

## Slide 1
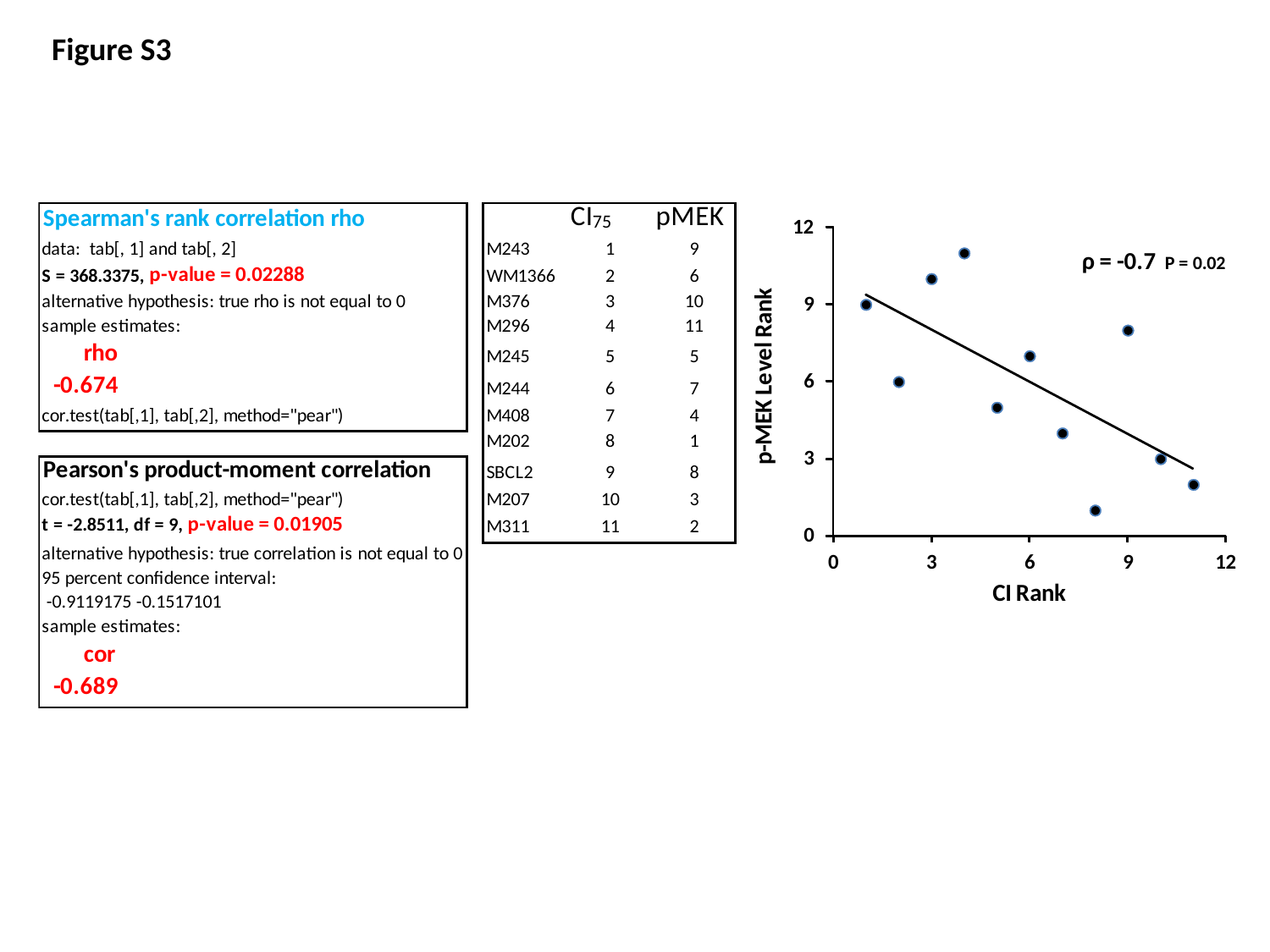

Figure S3

Supplement: Additional file 3: Figure S3. — CI75 and p-MEK level correlation plot in NRAS mutated cell lines (Spearman’s rank correlation analysis and Pearson’s product moment correlation). Ranking of CI75 of PRi + MEKi shows an inverse correlation with the ranking of p-MEK levels. [file 12943_2015_293_MOESM3_ESM.ppt]

## Slide 1
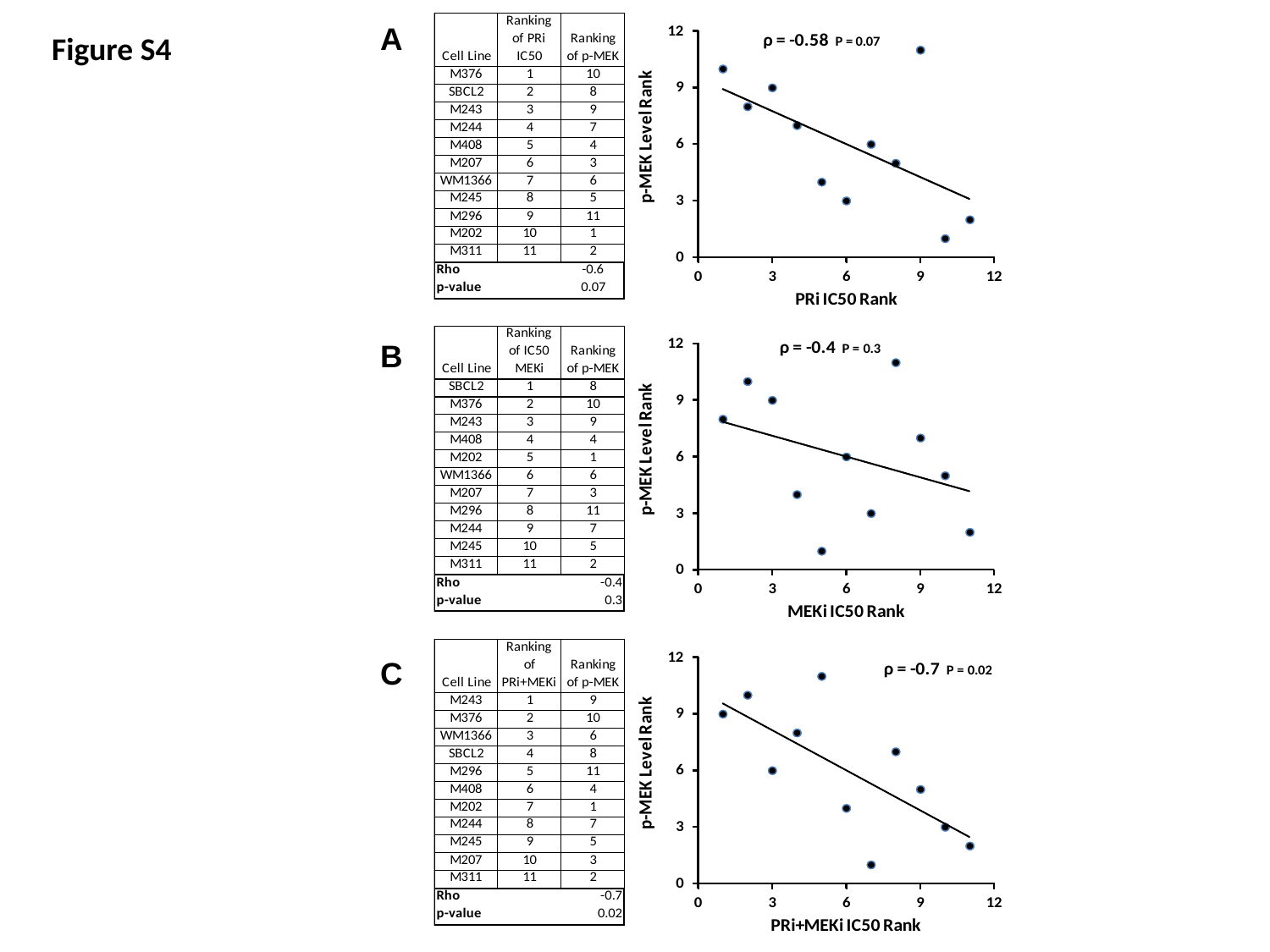

Figure S4

Supplement: Additional file 4: Figure S4. — IC50 and p-MEK level correlation plots in NRAS mutated cell lines (Spearman’s rank correlation analysis). Ranking of IC50s of PRi + MEKi shows an inverse correlation with the ranking of p-MEK levels. A) Correlation plot of ranking of PRi IC50s and ranking of p-MEK levels. B) Correlation plot of ranking of MEKi IC50s and ranking of p-MEK levels. C) Correlation plot of ranking of PRi + MEKi IC50s and ranking of p-MEK levels. [file 12943_2015_293_MOESM4_ESM.ppt]

## Slide 1
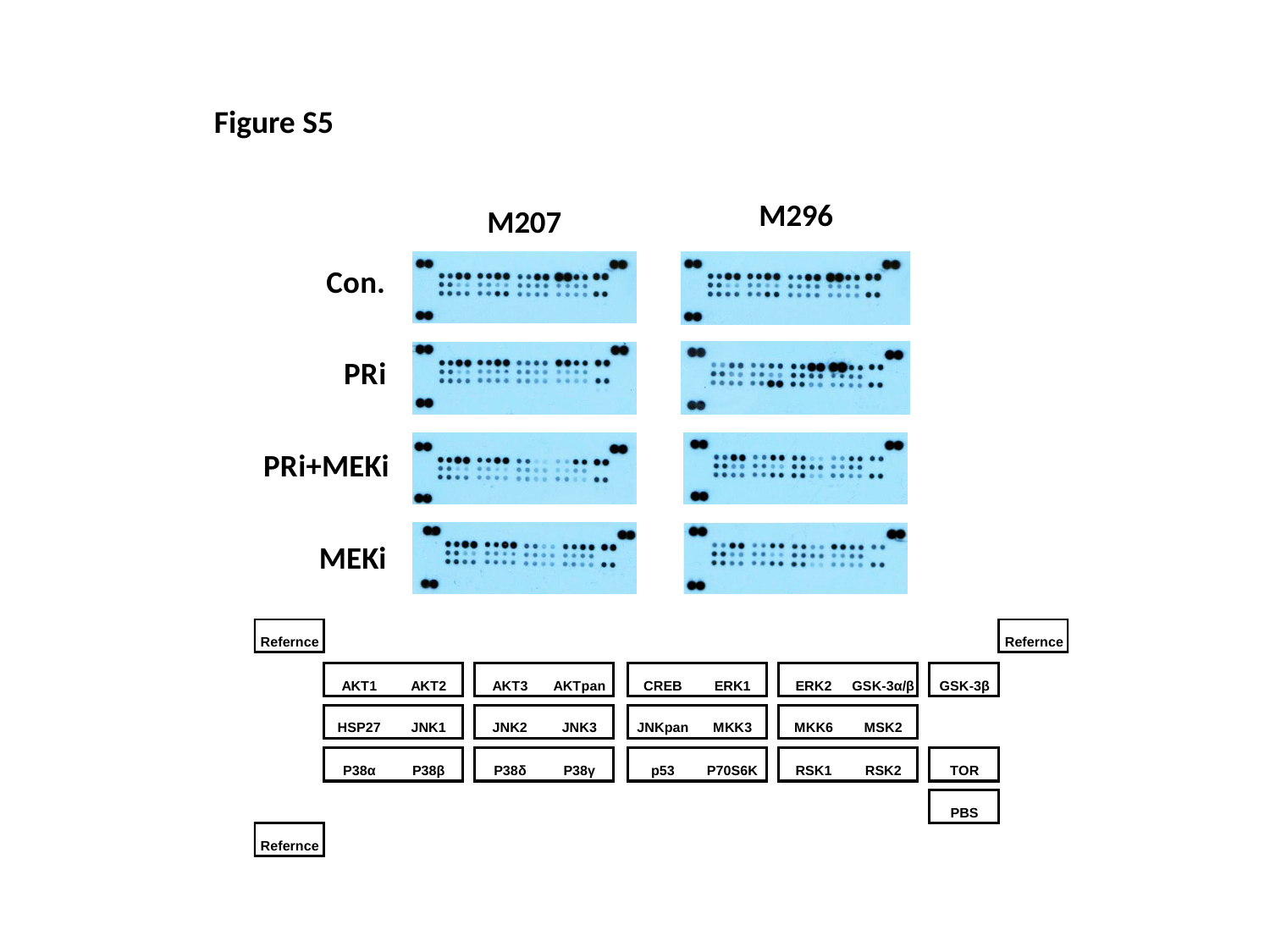

Figure S5

Supplement: Additional file 5: Figure S5. — Human Phospho-MAPK Array for detection of 24 phosphoproteins performed on melanoma cell lines. A) Differences in signaling of the resistant and sensitive cell lines M207 and M296 were investigated after treatment of these cell lines with the solvent, PRi (500 nM), MEKi (25 nM) and their combination for 24 hours. For this purpose the antibody array from R & D Systems was used which detects 24 phosphoproteins in duplicates. Each spot represent one phosphoprotein. The signals from the Reference spots were used for the normalization among the arrays. B) Map of the phosphoprotein spots on the array adopted from R & D systems website. [file 12943_2015_293_MOESM5_ESM.ppt]
